# A Novel *COMP* Mutated Allele Identified in a Chinese Family with Pseudoachondroplasia

**DOI:** 10.1155/2021/6678531

**Published:** 2021-03-08

**Authors:** Bing-Bing Guo, Jie-Yuan Jin, Zhuang-Zhuang Yuan, Lei Zeng, Rong Xiang

**Affiliations:** ^1^Department of Orthopaedics, Xiangya Hospital of Central South University, Changsha, China; ^2^School of Life Sciences, Central South University, Changsha, China; ^3^Hunan Key Laboratory of Animal Models for Human Diseases, School of Life Sciences, Central South University, Changsha, China

## Abstract

Pseudoachondroplasia (PSACH) is an autosomal dominant skeletal dysplasia with an estimated incidence of ~1/60000 that is characterized by disproportionate short stature, brachydactyly, joint laxity, and early-onset osteoarthritis. *COMP* encodes the cartilage oligomeric matrix protein, which is expressed predominantly in the extracellular matrix (ECM) surrounding the cells that make up cartilage, ligaments, and tendons. Mutations in *COMP* are known to give rise to PSACH. In this study, we identified a novel nucleotide mutation (NM_000095.2: c.1317C>G, p.D439E) in *COMP* responsible for PSACH in a Chinese family by employing whole-exome sequencing (WES) and built the structure model of the mutant protein to clarify its pathogenicity. The novel mutation cosegregated with the affected individuals. Our study expands the spectrum of *COMP* mutations and further provides additional genetic testing information for other PSACH patients.

## 1. Introduction

Pseudoachondroplasia (PSACH, OMIM 177170), whose incidence is estimated to be ~1/60000 (http://www.orpha.net/), is a relatively common osteochondrodysplasia. Its clinical features include disproportionate short stature, early-onset osteoarthrosis, spinal, epiphyseal and metaphyseal dysplasia, and loose joints [1]. Typically, the sufferer is normal at birth and usually arouses medical attention at ~2 years of age, as a wadding gait and diminished linear growth gradually appear[2].

Joint pain can begin in mid-childhood, particularly in the knees, hips, and ankles [3]. Vertebral anomalies usually gradually vanish with age. Milder cases of PASCH manifest similar radiographic features, but short-limbed dwarfism is less noticeable, and there is less deformity in milder cases of PASCH than in severe cases of PASCH [4]. However, PSACH patients generally have a normal craniofacial appearance and intelligence[2, 5].

PSACH is an autosomal dominant disease that is considered to result from mutations in the gene encoding the cartilage oligomeric matrix protein (COMP) [1, 6, 7]. The human *COMP* gene localizes on chromosome 19p13.1 and is a member of the thrombospondin gene family. It contains 19 exons, encoding an amino-terminal coiled-coil oligomerization domain, four type II epidermal growth factor-like repeats (EGF-like), eight type 3 calmodulin-like repeats (CLRs/T3 repeats), and a globular carboxyl terminal domain (CTD). It is abundantly expressed in the extracellular matrix (ECM) surrounding the cells that make up cartilage, ligaments, synovium, and tendons. Recent work suggests that COMP may stimulate chondrocyte proliferation by directly binding to the granulin/epithelin precursor (GEP) [8, 9]. Numerous *COMP* mutations have been identified in PSACH patients, and most of them were in the highly conserved T3 repeats[10, 11]. Point mutations resulting in single amino acid substitutions for conserved residues and in-frame deletions that delete codon(s) for one or more residues are the dominant incident [12].

Based solely on the family history, a detailed physical examination, and the radiographic method, it is difficult to accurately diagnose individuals with PSACH. For example, multiple epiphyseal dysplasia (MED, OMIM 132400) has semblable phenotypes and inherited patterns. With the application of next-generation sequencing techniques, such as whole-exome sequencing (WES), the discovery of the genetic architecture of individuals encountering heterogeneous conditions, such as PSACH, has extended, and it has also enabled the diagnosis of PSACH with more confidence[13].

In this study, we detected a novel mutated allele in *COMP* (NM_000095.2: c.1317C>G, p.D439E) in a Chinese family diagnosed with highly suspected PSACH based on clinical and radiologic results. To the best of our knowledge, there have been no reports of this variant in previous studies that have also not been presented in various single nucleotide polymorphism (SNP) databases.

## 2. Materials and Methods

### 2.1. Patients and Subjects

This research was approved by the Review Board of Xiangya Hospital of Central South University. Written informed consent was obtained from the proband and her guardians, in which all subjects consented to this study and the publication of images.

### 2.2. DNA Extraction

Peripheral blood samples of the proband and her family were collected to extract genomic DNA using the DNeasy Blood & Tissue Kit (Qiagen, Valencia, CA, USA).

### 2.3. Whole-Exome Sequencing

We followed the methods of Jin et al. [14] and Tang et al. [15]. The Berry Genomics Co., Ltd. (Chengdu, China) provided exome capture using the cBot Clster Generation System and HiSeq PE Cluster Kit (Illumina, San Diego, CA, USA) and high-throughput sequencing by the Illumina HiSeq 2500 platform (Illumina, San Diego, CA, USA). The common variants (frequency ≥ 0.05) were filtered according to the Genome Aggregation Database (GnomAD; http://gnomad.broadinstitule.org) and the 1000 Genomes Project database (1000G; https://www.genome.gov/27528684/1000-genomes-project/). Candidate disease-causing variants were screened by the skeletal dysplasia-related gene list (Table S1), and then, pathogenicity was predicted by MutationTaster software (http://www.mutationtaster.org/), the SIFT server (http://provean.jcvi.org/index.php), and Polymorphism Phenotyping v2 server (Polyphen-2; http://genetics.bwh.harvard.edu/pph2/). The annotation of inheritance patterns, clinical phenotypes, and gene functions was conducted by Online Mendelian Inheritance in Man (OMIM; https://www.omim.org).

### 2.4. Cosegregation Analysis

Primer pairs (COMP 5 ⟶ 3 f: GACAGCGATCAAGACCAGTAAG; COMP 5 ⟶ 3 r: CACACGTCGATCTTGTCTACC) were designed by Integrated DNA Technologies (https://sg.idtdna.com/pages). The target fragment was amplified by polymerase chain reaction (PCR) and analyzed using the ABI 3100 Genetic Analyzer (ABI, Foster City, CA, USA).

### 2.5. Modeling of the COMP Mutant

The structure of the COMP complex (PDB ID: 3FBY) was obtained from the Protein Data Bank in Europe database (ePDB; https://www.ebi.ac.uk/pdbe/?tdsourcetag=s_pcqq_aiomsg). PyMol was used to build the D439E mutant model according to the wild-type COMP structure.

## 3. Results

### 3.1. Patients' Characteristics

**Figure 1:**
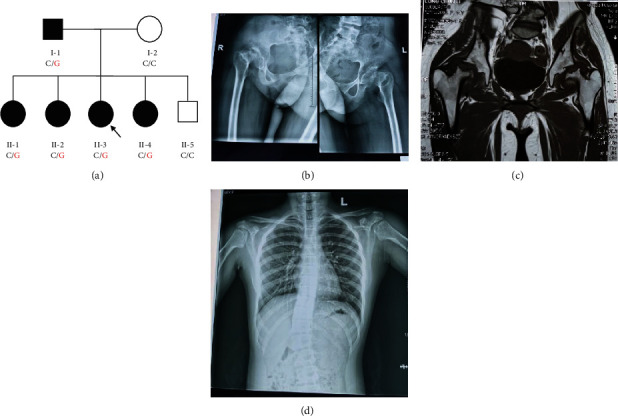
(a) Pedigree of a PSACH family with segregation analysis. The black symbols represent affected members, and arrows indicate the proband. Genotypes are identified by letters and a slash, with red representing mutations. (b–d) The phenotypes of the proband. The proband has congenital dysplasia of the hip (b, c) and scoliosis (d).

We enrolled a Chinese family with highly suspected PSACH (Figure 1(a)). The proband (II-3), a 14-year-old girl from Hunan Province of central south China with short stature (140 cm, <3%, no hormonal abnormalities), was primarily diagnosed with congenital dysplasia of the hip and required total hip replacement surgery after reaching an adult age. A medical history investigation revealed that the proband had suffered from wadding gait for ~12 years and bilateral hip joint pain for a year with aggravation for 5 months. Hip movement was restricted, particularly in flexion and abduction. The knees were positioned in the genu varum. Other studies with a positive Allis sign and Patrick sign suggested that the patient may have sacroiliac joint disease, but muscle tension was normal. Radiographs showed acetabular dysplasia with distorted acetabular shape, small epiphyses and femoral head deformity (Figures 1(b) and 1(c)). The right lower limb was shortened by 2 cm. The patient also had mild scoliosis with a Cobb angle of 17°, without brachydactyly and shoulder malformation (Figure 1(e)). Family history examination showed that her father (I-1, 155 cm) had necrosis of bilateral femoral heads and had undergone right hip replacement surgery in 2020. In addition, all of her sisters (II-1, II-2, and II-4) were short and presented unusual gait and hip dysplasia (Table 1). Her mother (I-1) and brother (II-5) were in good health.

### 3.2. Genetic Analysis

Common variants were filtered according to various SNP databases, and 776 unique SNPs were detected. After screening against a series of skeletal dysplasia causative genes, eight variants were identified in the proband (Table 2, Table S1). We classified these variants based on the American College of Medical Genetics (ACMG) guidelines [16] and strongly suspected that the heterozygous nucleotide variant of *COMP* (NM_000095.2: c.1317C>G, p.D439E) was the causative mutation in the family.

**Figure 2:**
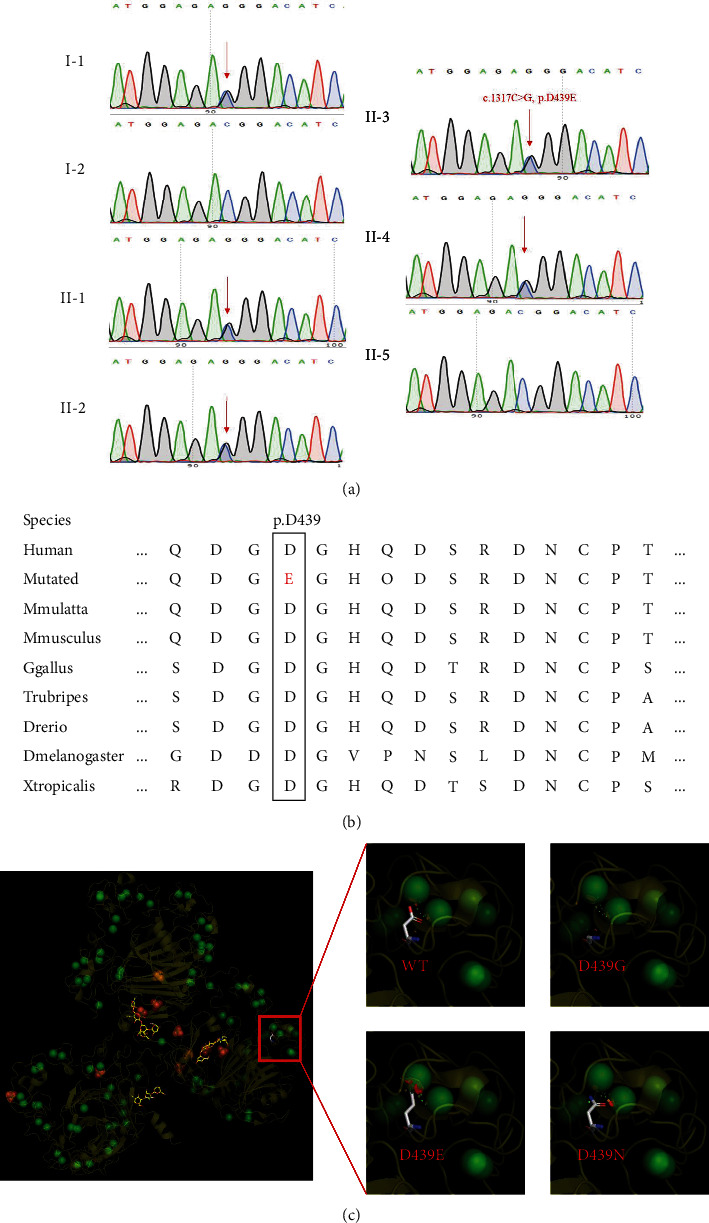
(a) The sequencing result of the *COMP* mutation. Sequence chromatograms indicate the heterozygous variant (c.1317C>G, p.D439E) in all affected members of this family. (b) The mutated site (D439E) is highly evolutionary conserved across species. The red graph represents mutated amino acids, and the black box emphasizes these sites across species for comparison. (c) The protein complex of COMP with or without mutants. Green balls represent Ca^2+^.

Sanger sequencing revealed that a novel mutated allele in *COMP* (c.1317C>G, p.D439E) was identified in the proband (II-3), which was inherited from her father (I-1), and her sisters (II-1, II-2, and II-4) also harbored this mutation (Figure 2(a)). The *COMP* mutation cosegregated with the affected family members. Cross-species alignment analysis of COMP showed that this mutated site had high conservation (Figure 2(b)). The D439E mutant model showed that compared with wild-type COMP, the mutant site broke a Ca^2+^-binding region. Therefore, we considered that the *COMP* mutation (c.1317C>G, p.D439E) was the pathogenesis of PSACH in this family.

## 4. Discussion

In this research, we used WES to identify a novel nucleotide variant (c.1317C>G, p.D439E) in exon 13 of *COMP* in a girl (II-3) with PSACH. Her father (I-1) and sisters (II-1, II-2, and II-4) carried the same mutation and had similar symptoms with an onset in early childhood. COMP is secreted by the endoplasmic reticulum of chondrocytes, and the misfolding of COMP inhibits its secretion, thereby causing toxicity to cells and leading to cell death[11, 17, 18]. Moreover, COMP has a significant effect on maintaining cartilage and ECM integrity, especially affecting the assembly of collagen fibers [11, 19]. There is a recent hypothesis that scoliosis with or without vertebral malformations may occur in association with arthrogryposis and can be triggered by defects in connective tissue matrix proteins. It was confirmed again in the proband and her young sister (II-3 and II-4), who harbored a *COMP* mutation and suffered from mild scoliosis and hip contractions.

COMP consists of a coiled-coil domain, four EGF-like repeats, eight T3 repeats, and a CTD domain. The mutations located at the T3 repeats region of COMP seem to cause a more severe phenotype. The T3 repeats region is a Ca^2+^-binding pocket[8]. A high proportion of specific residues, such aspartic acid, glycine, cysteine, and proline, in this region also promotes protein folding and Ca^2+^ binding [20]. In the D439E mutant, aspartic acid was substituted with glutamic acid, possibly reducing the stability of the mutant and inhibiting the binding ability of Ca^2+^. Mupro (https://www.ics.uci.edu/~baldig/mutation.html) predicted that D439E can decrease the stability (ΔΔ*G* = −0.7849), as expected. It has also been mentioned that chondrocyte attachment may be altered, for the three-dimensional Ca^2+^-dependent structure of mutant COMP get changed [21]. All of these functions are closely associated with the generation and development of PSACH. The present mutation (c.1317C>G, p.D439E) occurred on the T3 repeats domain, which may affect the structure and function of COMP, and our proband (I-3) was diagnosed with PSACH.

MED is also a skeletal dysplasia similar to PSACH, but with milder severity. MED patients present short statures (final adult height: 145-170 cm), hip osteoarthritis, mild genu varum, and mild irregularity of vertebral endplates [18]. It is difficult to distinguish PSACH and MED. Currently, most studies think that the biggest difference is that MED hardly leads to spine dysplasia [21, 22]. Most mutations of MED that have been reported involve *COMP* mutations. In addition to *COMP*, some forms of MED can also be caused by mutations in *MATN3*, *COL9A1*, *COL9A2*, *COL9A3*, *COL2A1*, *FGFR1*, *SLC26A2* and *DTDST* [23–25]. In our study, the proband was highly suspected of having PSACH based on the following points: (1) Only the *COMP* mutation was found as a possible disease-causing gene by WES and cosegregated with the affected family members. (2) The major phenotypes of the proband include short stature, gait wadding ,and early-onset osteoarthrosis, which are consistent with PSACH. Although vertebral anomalies usually resolved with age, mild scoliosis was also found in the patient. (3) Mutations in the T3 repeats domain of COMP were more likely to cause a severe phenotype in PSACH patients. In our case, the mutation (c.1317C>G, p.D439E) located at the 6^th^ repeat of T3 repeats (T3_6_), and according to the hypothesis of Briggs et al., missense mutations in T3_6_ had a greater frequency of PSACH than MED[20].

**Figure 3:**
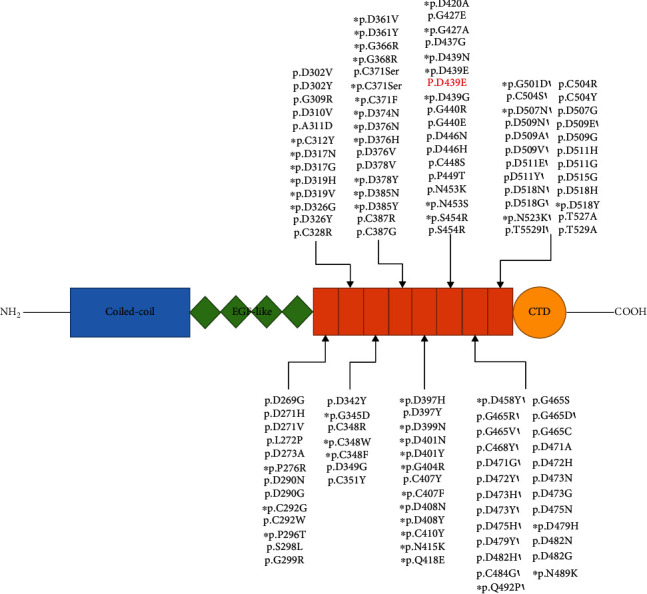
*COMP* mutations that occurred at the T3 repeats domain were identified in PSACH and MED patients. COMP has the NH_2_-terminus on the left and the COOH-terminus on the right. Known functional domains include an amino-terminal coiled-coil oligomerization domain (Coiled-coil), four type II epidermal growth factor-like repeats (EGF-like), eight type 3 calmodulin-like repeats (CLRs/T3 repeats), and a globular carboxyl terminal domain (CTD). Arrows show the approximate location of *COMP* mutations. The red words representing the present mutation. Asterisks represent these mutations were reported in MED cases, and others were identified in PSACH patients.

To data, there are at least 191 mutations in *COMP* that have been reported (http://www.hgmd.cf.ac.uk/ac/search.php). Among these mutations, the vast majority was connected with PSACH and was mostly located in the T3 repeats domain (Figure 3). Thus, the domain is a mutation hot spot, where the *COMP* mutation we identified was located. In addition to a large number of missense mutations, it also includes in-frame small deletions, insertions, or indels [12, 26, 27]. Three *COMP* mutations (c.1315G>A, p.D439N; c.13169r, p.D439G; c.1317C>A, p.D439E) were identified in MED cases, which altered the 439^th^ amino acid (AA) of COMP[20, 28, 29]. These mutations indicated the pathogenicity of AA alterations, and the present mutation (c.1317C>G, p.D439E) also occurred at this AA site. By analyzing these three mutant protein models, we considered that D439N and D439G alter the electric charge of a Ca^2+^-binding site and that D439E breaks the spatial configuration of this binding region (Figure 2(c)). Unlike MED cases with these three known mutations, our proband had PSACH. This may be caused by ethnic or individual differences. In fact, many researchers thought that these two malformations should be classified as the same disease with different severities. Interestingly, it seems that these mutations are more likely to disrupt the folding and tertiary structure of COMP[20]. Studies in *COMP* knocked out mice had suggested that it is not a reduction in COMP but a dysfunctional mutated COMP that results in PSACH[22, 30].

Actually, the diagnosis of PSACH mostly relies on patients' characteristics, family history, clinical symptoms, and radiological features [31]. There is currently no appropriate therapy for this genetic disease, and only symptomatic treatments are available [32]. Moreover, the boundary between PSACH and MED is indefinite. Recently, with the application of next-generation sequencing techniques, such as WES, the discovery of the genetic pathogenetic factor of individuals in heterogeneous conditions has facilitated the diagnosis of skeletal disorder disease, and it is also a powerful and cost-efficient method to verify the causative gene [13, 26]. In the future, further efforts will be made to provide assistance in genetic counseling and disease prognosis.

## 5. Conclusions

In conclusion, we detected a novel nucleotide variant (NM_000095.2: c.1317C>G, p.D439E) in *COMP* in a proband diagnosed with mild PSACH. Our study expands the spectrum of COMP mutations. Although we still know little about the exact mechanism and progression of the disease, this data may contribute to genetic diagnosis and counseling of families with PSACH.

## Figures and Tables

**Table 1:** The clinical symptoms of patients in the present PSACH family.

Patients	Sex	Age (years)	Height (cm)	Onset ages (years)	Gait abnormalities	Hip dysplasia	Knee contractions	Scoliosis	Brachydactyly	Myasthenia
I-1	M	51	155	Unknown	+	+	+	—	—	—
II-1	F	17	145	3	+	+	Unknown	Unknown	—	Unknown
II-2	F	15	140	2	+	+	+	—	—	Unknown
Proband	F	14	140	2	+	+	+	+	—	—
II-4	F	12	135	3	+	+	—	+	—	Unknown

M: male; F: female; +: positive phenotype; -: negative phenotype.

**Table 2:** Variants identified by WES in combination with skeletal dysplasia-related gene-filtering in the proband.

Gene	Mutation	Mutation taster	PolyPhen-2	SIFT	1000G	gnomAD	OMIM clinical phenotype	ACMG classification
GNPTAB	c.673C>A, p.Q225K	D	B	T	—	0.00000	AR, mucolipidosis 2 alpha/beta; AR, mucolipidosis 3 alpha/beta.	BS (PM2, BS4, BP4, BP5)
TRPV4	c.2569C>A, p.Q857K	P	B	T	—	0.00002	AD, brachyolmia type 3; AD, digital arthropathy-brachydactyly, familial; AD, hereditary motor and sensory neuropathy, type 2c; AD, scapuloperoneal spinal muscular atrophy; AD, SED, Maroteaux type.	BP (BP4, BP5)
TGFB1	c.77C>T, p.P26L	D	B	T	—	—	AD, Camurati-Engelmann disease; AR, inflammatory bowel disease, immunodeficiency, and encephalopathy.	BP (PM2, BP4, BP5)
FLNB	c.2671G>A, p.D891N	P	B	T	—	0.00004	AD, atelosteogenesis, type 1/3; AD, boomerang dysplasia; AD, Larsen syndrome; AR, spondylocarpotarsal synostosis syndrome.	BP (BP4, BP5)
COL9A1	c.674A>T, p.D225V	D	B	D	0.00040	0.00025	AD, epiphyseal dysplasia, multiple, 6.	BP (BP4, BP5)
RECQL4	c.1396C>A, p.P466T	D	B	T	—	—	AR, Baller-Gerold syndrome; AR, Rothmund-Thomson syndrome, type 2.	BS (PM2, BS4, BP4, BP5)
*COMP*	*c.1317C>G, p.D439E*	*D*	*D*	*D*	*—*	*—*	*AD, epiphyseal dysplasia, multiple, 1; AD, pseudoachondroplasia.*	*PS (PS1, PM1, PM2, PP1, PP3, PP4)*
EVC	c.769_772delinsTTAC, p.Y258H	P	B	T	—	—	AD, Weyers acrofacial dysostosis; AR, Ellis-van Creveld syndrome.	BP (PM2, BP4, BP5)

Italicized words: mutations identified in this study; D: disease causing; B: benign; T: tolerated; P: polymorphism; AR: autosomal recessive; AD: autosomal dominant. Pathogenic: PVS1> PS1>…> PS4> PM1-6> PP1-5; benign: BA1> BS1-4> BP1-7. PVS: pathogenic very strong; PS: pathogenic strong; PM: pathogenic moderate; PP: pathogenic supporting; BA: benign stand-alone; BS, benign strong; BP, benign supporting.

## Data Availability

The datasets used and/or analyzed during the current study are available from the corresponding author upon reasonable request.

## Supplementary Materials

Supplementary Materials For supplementary material file “Table S1”: we collected 224 skeletal dysplasia-related genes as candidate genes of the proband's disease, and we made a list as a supplementary table.
