# Supplementary material for: A Novel COMP Mutated Allele Identified in a Chinese Family with Pseudoachondroplasia
Source: Biomed Res Int. 2021 Mar 8;2021:6678531. doi: 10.1155/2021/6678531 (PMC7960025; doi:10.1155/2021/6678531)
Supplement: Supplementary Materials — For supplementary material file “Table S1”: we collected 224 skeletal dysplasia-related genes as candidate genes of the proband's disease, and we made a list as a supplementary table. [file 6678531.f1.docx]

**Supplementary Table 1.** The skeletal dysplasia-related genes list.

| *ACVR1* | *CASR* | *COMP* | *EIF2AV3* | *FUCA1* | *HOXA13* | *LMNA* | *OBSL1* | *RAB23* | *SLC17A5* | *TRAIP* |
| --- | --- | --- | --- | --- | --- | --- | --- | --- | --- | --- |
| *ADAMTS10* | *CBFB* | *CRTAP* | *EIF4A3* | *GALNS* | *HOXD13* | *LMX1B* | *ORC1* | *RANK* | *SLC34A3* | *TRAP* |
| *ADAMTSL2* | *CBP* | *CTSA* | *ENPP1* | *GDD1* | *HPGD* | *LRP5* | *ORC4* | *RANKL* | *SMAD4* | *TRIP11* |
| *AGA* | *CCDC8* | *CTU2* | *EP300* | *GDF3* | *HSPG2* | *MAN2B1* | *OSTM1* | *RBM8A* | *SMARCAL1* | *TRPS1* |
| *AGC1* | *CDC6* | *CUL7* | *ESCO2* | *GDF5* | *HSS* | *MANBA* | *OSX* | *RECQL4* | *SMC1L1* | *TRPV4* |
| *AGPS* | *CDH3* | *CWC27* | *EVC* | *GDF6* | *IDS* | *MATN3* | *PAPSS2* | *RMRP* | *SMC3* | *TWIST1* |
| *ALPL* | *CDPX1* | *CXORF5* | *EVC2* | *GJA1* | *IDUA* | *MESP2* | *PCNT* | *RNU4* | *SNRPB* | *TXNL4A* |
| *ALX1* | *CDT1* | *DHCR24* | *EXT1* | *GLB1* | *IFITM5* | *MGP* | *PDE4D* | *ROR2* | *SNX3* | *WDR35* |
| *ALX3* | *CHST3* | *DHODH* | *EXT2* | *GLI3* | *IFT122* | *MMP13* | *PEX7* | *Runx2* | *SOST* | *WISP3* |
| *ALX4* | *CIAS1* | *DLL3* | *FAM20C* | *GNAS1* | *IHH* | *MMP2* | *PHEX* | *SALL1* | *SOX9* | *WNT3* |
| *ANKH* | *CLCN7* | *DLX3* | *FBN1* | *GNAT* | *KAT6B* | *MMP9* | *PLEKHM1* | *SALL4* | *SUMF1* | *WNT5A* |
| *ARHGAP31* | *CMG2* | *DLX4* | *FGF23* | *GNPAT* | *KIF22* | *MSX2* | *PLOD2* | *SBDS* | *TBCE* | *WNT7A* |
| *ARSB* | *COL10A1* | *DLX5* | *FGF9* | *GNPTAB* | *KIF7* | *NAGLU* | *POLR1C* | *SCYL1BP1* | *TBX3* | *WTX* |
| *ATAC* | *COL11A1* | *DMP1* | *FGFR1* | *GNPTG* | *LAF4* | *NEK* | *POLR1D* | *SEDL* | *TBX4* | *ZMPSTE24* |
| *B3GAT3* | *COL11A2* | *DOCK6* | *FGFR10* | *GNS* | *LBR* | *NEU1* | *PPIB* | *SERPINF1* | *TBX5* |  |
| *B4GALT7* | *COL1A1* | *DTDST* | *FGFR2* | *GPC6* | *LEMD3* | *NIPBL* | *PPM1D* | *SERPINH1* | *TBXAS1* |  |
| *BMP1* | *COL1A2* | *DYM* | *FGFR3* | *GUSB* | *LEPRE1* | *NOG* | *PRC6* | *SF3B4* | *TCIRG1* |  |
| *BMP2* | *COL2A1* | *DYNC2H1* | *FKBP10* | *GZF1* | *LFNG* | *NOTCH2* | *PRKAR1A* | *SH3BP2* | *TCOF1* |  |
| *BMPR1B* | *COL9A1* | *EBP* | *FKBP65* | *HES7* | *LGI4* | *NPR2* | *PTHR1* | *SHH* | *TGFB1* |  |
| *CAII* | *COL9A2* | *EFNB1* | *FLNA* | *HLXB9* | *LIFR* | *NR2E1* | *PTPN11* | *SHOX* | *TNFRSF11B* |  |
| *CANT1* | *COL9A3* | *EFTUD2* | *FLNB* | *HOXA11* | *LMBR1* | *NSDHL* | *PYCR1* | *SHOXY* | *TP63* |  |
